# The Aging Enterprise: A 40-Year Retrospective

**DOI:** 10.1093/geroni/igaa057.2537

**Published:** 2020-12-16

**Authors:** Larry Polivka, Carroll Estes

## Abstract

Dr. Carroll Estes has long been recognized as one of our most influential social gerontologists beginning with the publication of the Aging Enterprise over 40 years ago. This book quickly achieved iconic status among gerontologists and other social scientists as one of the founding texts in critical gerontology, which Dr. Estes has played a leading role in developing with numerous publications over the course of her illustrious career. The panelists will focus on Dr. Estes’ application of the theoretical frameworks offered by the social construction of reality and the political economy of aging to a critique of federal and state policies designed to improve the quality of life of older Americans. Many of the programs and policies included in Dr. Estes’ critique are still in place, including the Older Americans Act and the nonprofit aging network. On the other hand, much about the aging enterprise has changed since 1979. The panelists, Drs. Chris Phillipson, Pamela Herd and Larry Polivka, will discuss the value of and challenges to these theoretical and empirical perspectives within the current contemporary neoliberal political economy that has gradually displaced the welfare state capitalism of the postwar period. As this shift has occurred in the political economy, a neoliberal policy agenda featuring for-profit privatization of public services, including aging services, has become dominant at the federal and state levels. Dr. Estes will respond to the panelists’ presentations and discuss the future of critical gerontology. Women's Issues Interest Group Sponsored Symposium.

